# Conformational Response to Solvent Interaction and Temperature of a Protein (Histone h3.1) by a Multi-Grained Monte Carlo Simulation

**DOI:** 10.1371/journal.pone.0076069

**Published:** 2013-10-18

**Authors:** Ras B. Pandey, Barry L. Farmer

**Affiliations:** 1 Department of Physics and Astronomy, University of Southern Mississippi, Hattiesburg, Mississippi, United States of America; 2 Materials and Manufacturing Directorate, Air Force Research Laboratory, Wright Patterson Air Force Base, Dayton, Ohio, United States of America; Russian Academy of Sciences, Institute for Biological Instrumentation, Russian Federation

## Abstract

Interaction with the solvent plays a critical role in modulating the structure and dynamics of a protein. Because of the heterogeneity of the interaction strength, it is difficult to identify multi-scale structural response. Using a coarse-grained Monte Carlo approach, we study the structure and dynamics of a protein (H3.1) in effective solvent media. The structural response is examined as a function of the solvent-residue interaction strength (based on hydropathy index) in a range of temperatures (spanning low to high) involving a knowledge-based (Miyazawa-Jernigan(MJ)) residue-residue interaction. The protein relaxes rapidly from an initial random configuration into a quasi-static structure at low temperatures while it continues to diffuse at high temperatures with fluctuating conformation. The radius of gyration (*R_g_*) of the protein responds non-monotonically to solvent interaction, i.e., on increasing the residue-solvent interaction strength (*f_s_*), the increase in *R_g_* (*f_s_*≤*f_sc_*) is followed by decay (*f_s_*≥*f_sc_*) with a maximum at a characteristic value (*f_sc_*) of the interaction. Raising the temperature leads to wider spread of the distribution of the radius of gyration with higher magnitude of *f_sc_*. The effect of solvent on the multi-scale (*λ:* residue to *R_g_*) structures of the protein is examined by analyzing the structure factor (*S(* ***q*** *)*,*|q| = 2π/λ* is the wave vector of wavelength, *λ*) in detail. Random-coil to globular transition with temperature of unsolvated protein (H3.1) is dramatically altered by the solvent at low temperature while a systematic change in structure and scale is observed on increasing the temperature. The interaction energy profile of the residues is not sufficient to predict its mobility in the solvent. Fine-grain representation of protein with two-node and three-node residue enhances the structural resolution; results of the fine-grained simulations are consistent with the finding described above of the coarse-grained description with one-node residue.

## Introduction

A solvent medium is critical in controlling the structure and dynamics of a protein and to the performance of its specific function [1]–[10] (list of references is too large to cite). How the solvent affects the thermodynamic properties of a protein depends on the type of solvent, specificity of the protein, and temperature, among other variables, such substrate and protein concentration. For example, Hinsen and Kneller [4] have performed a molecular dynamics simulation on solvated and unsolvated lysozyme. Using the mode analysis they found that ‘solvent effects are important for the slowest motions but negligible for faster motion’ in low and high frequency modes, respectively. Xu et al [5], have performed MD simulation on a protein, E6ap, in water and trifluoroethanol with adaptive hydrogen bond-specific charge. From the analysis of the free energy, they found that ‘the solvent may determine the folding clusters of E6ap, which subsequently leads to the different final folded structure’. Kurkal et al. [3] have examined the effect of temperature and hydration on the low frequency enzyme (pig liver esterase) dynamics via neutron scattering. They found that ‘increasing hydration results in lower flexibility of the protein at low temperatures and increased flexibility at higher temperatures’ and that the interaction between the protein and the underlying environment is temperature dependent. Temperature dependence of the mean square displacement (MSD) of a protein (cytochrome P450cam) in powder form and water and that of its residues has been recently studied by Miao et al. [1] using elastic incoherent neutron scattering and molecular dynamics simulations. They found that ‘with increasing temperature, first the hydrophobic core awakens followed by the hydrophilic surface’. The main conclusion [1] of this study is, at low temperatures ‘protein flexibility arises from the hydrophobic and aromatic residues, which are dynamically activated, in contrast to the hydrophilic residues, the dynamics of which are suppressed as a result of stable hydrogen bonding interactions with the neighboring protein residues and hydration water’. Increasing the temperature leads to hydration-dependent transition in jumps of the hydrophilic group. Apparently, the local movement of residues and subsequent structures depend on the type of residues and their sequence, solvent and temperature.

Interplay between the cooperative and competing effect of residue-solvent and residue-residue interactions and temperature on the covalently bonded residues in a protein leads to interesting structural and dynamical response. Investigation of such a protein involves multiple scales, i.e., local self-assembly to global structures. Very recently, we have examined [11], [12] the conformation and dynamics (local and global) of unsolvated proteins as a function of temperature with a coarse-grained simulation. In this article, we examine the effect of solvent (via effective medium) on structure and dynamics of a protein (histone H3.1) as a function of temperature with a coarse-grained Monte Carlo simulation [11].

In recent years, we have been studying [11], [12] the structure and dynamics of unsolvated histones, which are core components of the nucleosome in eukaryotic cells. In coordination with a number of host constituents, histones direct the morphology of DNA (extended structure to condensed phase) by wrapping around and controlling its exposure to cellular machinery. Histones are thus involved at various stages in triggering specific response to collective action of the cell in both interphase (duplication of DNA) and mitosis (cell division).There are numerous proteins in the family of histones that perform specific functions in a coordinated fashion. For example, histone h3.1 is believed to undergo enormous structural changes in S-phase of the interphase and becomes highly modified in the post-translational state in order to direct the conformational changes of the DNA. The collective response emerges from the cooperative actions of a rather complex set of interacting components in the nucleus. Investigating the cooperative response properties of such interactive components requires understanding of each constituent first. The structure of an unsolvated histone, h3.1, exhibits a continuous conformational crossover [11] at a transition temperature (*T_c_*) from a random coil (at *T≥T_c_*) to a globular structure (at *T≤T_c_*) in a characteristic temperature range. Continuous transition with a rather constant size measured by its radius of gyration at both high (*T>T_c_*) and low (*T<T_c_*) temperatures appears to be unique characteristics [11], [12] of this protein in an idealized empty host space. Most structural changes of proteins however occur in a solvent (*in vitro* or *in vivo*) environment that plays a crucial role in controlling both conformation as well as dynamics. Therefore, we would like to investigate the effect of solvent interaction on the structure of the histone h3.1 with a specific sequence of 136 residues ^1^M^2^A^3^R…^136^A [11].

## Model and Methods

We consider a coarse-grained model [11] of the protein chain on a cubic lattice where a residue is represented by a node (the unit cell of the cubic lattice). The histone h3.1 is represented as a chain of 136 residues tethered together in a specific sequence via fluctuating covalent bonds on a cubic lattice. We also use fine-grain representations in which a residue is represented by two and three consecutive nodes. The number of nodes of the protein chain is accordingly increased, i.e. histone h3.1 consists of 272 and 408 nodes respectively in our fine-grain (two-node and three-node residue) representation as a result. The empty lattice sites constitute an effective solvent medium [8]. Specificity of each residue is incorporated via their unique residue-residue interactions as well as residue-solvent (empty site) interactions.

### Interactions

Each residue interacts with neighboring residues and solvent sites within a range (*r_c_*) with a generalized Lennard-Jones potential,
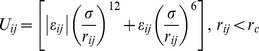
where *r_ij_* is the distance between the residues at site *i* and *j* or between the residue at site *i* and solvent at site *j*; *r_c_ = √8* and *σ = 1* in units of lattice constant. Note that the range of interaction includes lattice sites (solvent and residue) of the order of 100. The degree of freedom is enhanced vastly with our fine-grain representation of the protein chain. The potential strength *ε_ij_* is unique for each interaction pair with appropriate positive (repulsive) and negative (attractive) values. A knowledge-based interaction matrix [13] is used for the residue-residue pair interaction (*ε_ij_*), which is derived from an ensemble of a large number of protein structures from the protein data bank (PDB). A number of such interaction tables [14]–[17] are frequently used in investigating a range of issues related to protein structure. We resort here to the classic interaction table^13^ that was recently employed in a similar study [11] as well as in investigating scaffolding of short peptides.

The interaction between a residue (at a site *i*) and a solvent site (*j*) is based on the hydropathy index of each residue, *ε_ij_ = f_s_ ε_i_*. The empirical parameter *f_s_* can be varied to modulate the solvent quality, which could be considered as a measure of relative solvent pH; in this article, we also refer to it as residue-solvent interaction strength. The interaction *ε_i_* of a residue with the solvent sites is unique and depends on its hydropathy index [6]. The residue-solvent interaction [6] is thus positive (repulsive) for hydrophobic (*H*) residues and negative (attractive) for polar (*P*) and electrostatic (*E*) residues; the magnitude of *ε_i_* of a residue varies within each group (*H, P, E*) according to its relative hydropathy index.

### Unit and degrees of freedom

We use arbitrary units to analyze the changes in structures and identify patterns of the physical quantities (see below) in response to solvent interactions at various temperatures. Mapping of the arbitrary units to laboratory scales would be premature with our coarse-grained approach for two reasons, (*i*) the laboratory samples are much more complex than those used in the idealized computer simulation models and (*ii*) calibration of the parameters used in simulation requires measurements of some common physical quantities in both computer simulations as well as laboratory experiments which is not feasible at present. The response of the physical quantities, i.e., changes in patterns to parameters (e.g., interaction strength *f_s_* and temperature) could be compared qualitatively wherever feasible. It must be pointed out that there is a vast amount of work (the list is too large to cite them all here) on protein modeling using a rather diverse range of approximations from all-atom details to minimalist coarse-grained descriptions and tools entailing strength and weaknesses. Although we use a discrete lattice similar to minimalist methods, our approach provides ample degrees of freedom for each residue (one node) of the protein chain to execute its movements with variable covalent bond lengths (see below), many times more than the minimalist approach with fixed bond length. The degrees of freedom are increased enormously (about two to three fold) with our fine-grained approach with two-node and three node residue representations. We are able to explore large-scale thermodynamic properties of such a complex system due to the efficiency and effectiveness of such a coarse-grained approach.

### Stochastic moves

The protein chain is immersed in effective solvent medium where each tethered residue performs its stochastic motion with the Metropolis algorithm [18], [19] as follows. A residue, say at a site *i*, is selected randomly to move to one of its randomly selected neighboring lattice sites *j*. The excluded volume constraints and the limitations on changes in the covalent bond length *l* (*2≤l≤√10* with an exception of *√8* [19]) are checked and strictly implemented first. If these physical constraints are satisfied, then the attempt is made to move the residue from site *i* to site *j* with the Boltzmann probability *exp(−ΔE_ij_/T)*, where *ΔE_ij_* is the change in energy between its new (*E_j_*) and old (*E_i_*) configuration *ΔE_ij_ = E_j_−E_i_*; *T* is the temperature in reduced units of the Boltzmann constant and the energy (*ε_ij_*). As usual, attempts to move each residue once define the unit Monte Carlo step (MCS) time [18], [19].

### Quantities

During the course of simulations, we monitor a number of local and global physical quantities, e.g., the energy of each residue, its mobility, mean square displacement of the center of mass of the protein, radius of gyration, and its structure factor. Simulations are carried out for a sufficiently long time (typically for ten million time steps) at each temperature for a range of solvent interaction strength with many independent samples (typically *150* samples for long runs and 1000 samples for short runs) to evaluate the statistical averaging of these quantities. Different lattice sizes are used to verify that there is no finite size effect on the qualitative variations of the physical quantities and our conclusions; we constrain here to data generated on a *64^3^* lattice in coarse-grained representation of the protein chain with one-node residue. Larger lattices,*100^3^* and *210^3^*, are used with fine-grain representation (two-node and three-node residue) of the protein chain with 272 and 408 nodes respectively.

## Results and Discussion

**Figure 1:**
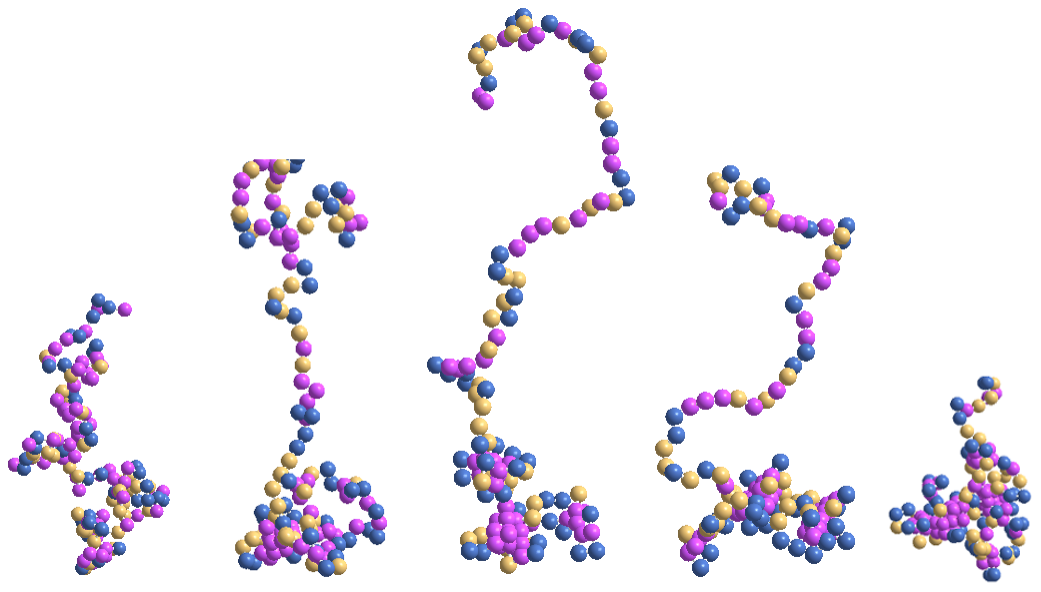
Snap shots of the protein at the end of *10^7^* time steps at temperature *T = 0.020* for the solvent interaction strength *f_s_ = 1, 5, 14, 16*, and *20* (from left to right) on a *64^3^* lattice. Colors pink (hydrophobic residues), golden (polar), and blue (electrostatic) represent residues in different groups without distinction within.

Most of the data presented below are based on the coarse-grained representation of the protein chain with one-node residue except towards the end where results from the fine-grained approach are included for comparison. A set of typical snapshots of the histone configurations at the end of *10^7^* time steps is presented in figure 1 for a range of solvent interaction strengths *f_s_ = 1–20* at a temperature *T = 0.020*. Obviously, a single configuration from a huge ensemble does not provide an estimate of the average morphology over the observable time span. These snapshots nevertheless show some important structural features regarding residue assembly across the protein length; animations provide better insight into the structural evolution in time.

A first glance at the snapshots reveals that the structure of the protein spreads and then contracts on increasing the interaction strength, i.e., a non-monotonic dependence of the radius of the protein on the solvent interaction. Such an observation can be quantified by analyzing appropriate physical quantities such as radius of gyration (see below). It is interesting to note how the self-assembly of residues occurs around specific segments. Size of the aggregates (globules) varies with the solvent interaction strength *f_s_*, i.e., relatively smaller sizes (with different shapes) at both low and high values of *f_s_*. In addition to self-assembly of the residues, the protein chain exhibits a wide variation in segmental morphology involving linear chains and loops. The competition between the residue-residue and residue-solvent interactions and the temperature leads to unique cooperative response with a rich ensemble of configurations. Such a unique yet versatile set of conformations plays a critical role in their assembly and directing the structure of DNA in the nucleosome and its exposure in the cell nucleus with evolving solvent medium.

### Energy and mobility profile

**Figure 2:**
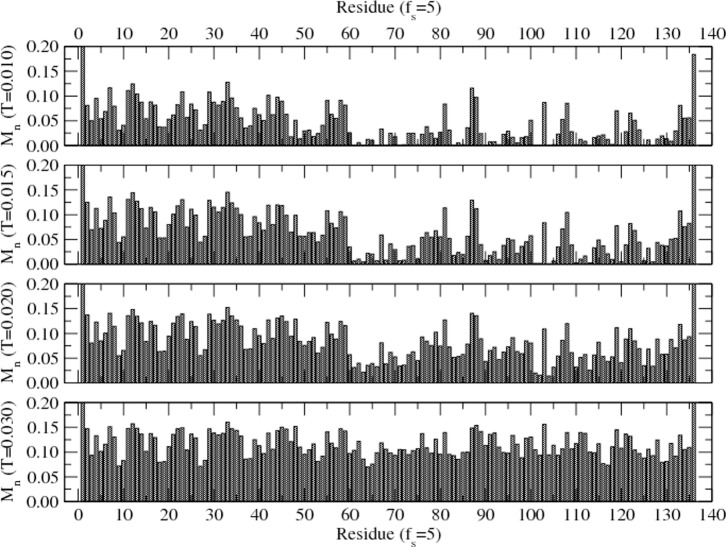
Average energy of each residue (of H3.1 protein) immersed in solvent (with *f_s_ = 5*) at temperatures T = 0.010–0.030. The interaction energy of each residue with its surrounding solvent and other residues within the range (*r_c_*) of interaction is evaluated at each time step but only its asymptotic (i.e., equilibrium) values are used in averaging. Simulations are performed on a 64^3^ lattice for 10^7^ time steps with 100 independent samples at each temperature and solvent strength.

Energy and mobility profiles of the protein residues are analyzed in detail for a range of solvent quality at different temperatures (low to high). Figure 2 shows the energy profile of the residue in equilibrium in a specific solvent medium at different temperatures (T = 0.010–0.030). Equilibrium is assessed by monitoring the approach of both the global energy of the protein and its radius of gyration to its asymptotic values. Interaction energy (*E_n_*) of each residue with the surrounding solvent sites and other residues within the range of interaction is estimated in equilibrium. Data for the energy during the later half of the simulation time steps are used in averaging in each independent sample.

The interaction energy of most residues appears to decrease on increasing the temperature in contrast to general expectation, although the energy of some residues shows an increase. Since each residue interacts with the surrounding solvent sites and residues with specific interactions, its energy depends on configurations evolved in equilibrium. For example, the energy *E_n_* of ^85^F (positive value in figure 2) increases while that of ^86^Q decreases on raising the temperature. We see that the energy profile of a protein with specific sequence is unique at each temperature in a specific solvent. Understanding the response properties of such a protein is complex due to such specificity that provides the versatility in its specific and global function.

**Figure 3:**
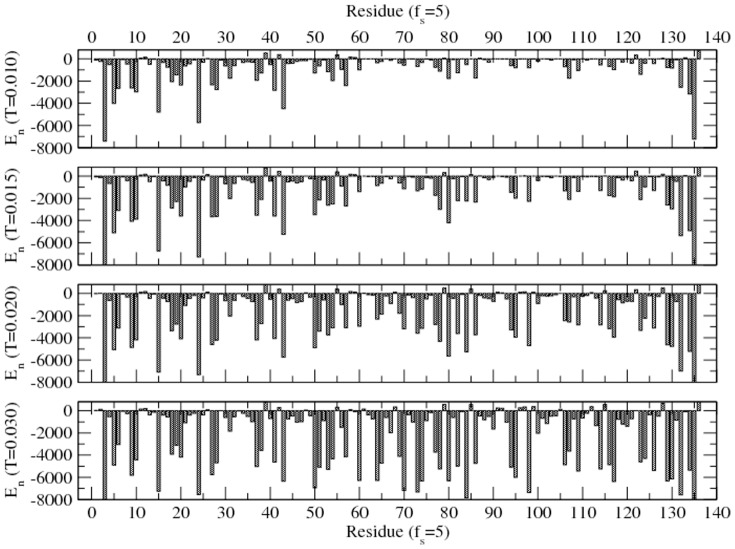
Average mobility (fraction of successful moves per unit time step) of each residue (of H3.1 protein) immersed in a solvent with the interaction strength *f_s_ = 5* at temperatures *T = 0.010–0.030* (corresponding to figure 2). Simulations are performed on a 64^3^ lattice for 10^7^ time steps with 100 independent samples at each temperature and solvent strength.

The dynamics of the protein are governed by the collective movement of each residue. How fast a residue moves depends on the type of residue and its position in sequence, temperature, and the local environment (i.e., interacting solvent and residue and constraining covalent bond) in which it is embedded. Average number of moves per unit time steps is defined as mobility; but it is actually a measure of a residue's stochastic movement. A typical mobility profile of the residues in a solvent (*f_s_ = 5*) at different temperatures (corresponding to energy profile of figure 2) is presented in figure 3.

The mobility of the residues at the ends (^1^M, ^136^A) is the highest due to their lesser covalent constraints. Therefore the mobility of the interior residues is a better measure of the internal dynamics of the protein. At the low temperature (e.g., T = 0.010), about half of the residues (in segment ^61^L-^135^R) are nearly frozen with spikes in their mobility (e.g., 81T, 86Q, ^103^G, ^108^T, ^119^T, ^133^G). Raising the temperature (e.g., T = 0.015) enhances their mobility somewhat. At high temperatures, almost all residues perform their stochastic movements at about the same frequency.

An increase in temperature leads to an increase in mobility of all residues, however the rate of increase in mobility at high temperatures is the most for those residues that are least mobile at low temperatures. Note that the mobility of all interior residues (constrained by covalent bonds) never approaches that of the end residues (least constrained). Apart from the constraints imposed by the covalent bond, segmental self-assembly due to non-covalent interaction adds further constraints (local caging) at lower temperatures (T = 0.010–0.020). At high enough temperatures (T≥0.030), the interaction becomes least dominant, the protein behaves as a polymer chain losing its specificity. It should be pointed out that the energy alone is not the only measure in assessing the stability of intra-chain assembly. For example, the interaction energies of residues ^85^F and ^86^Q show opposite trends (increasing and decreasing) with increasing the temperature while both residues continue to become more mobile. The activation of protein is thus limited to a certain temperature range dictated by the residue-residue and residue-solvent interactions in a somewhat complex fashion.

### RMS displacement and radius of gyration

**Figure 4:**
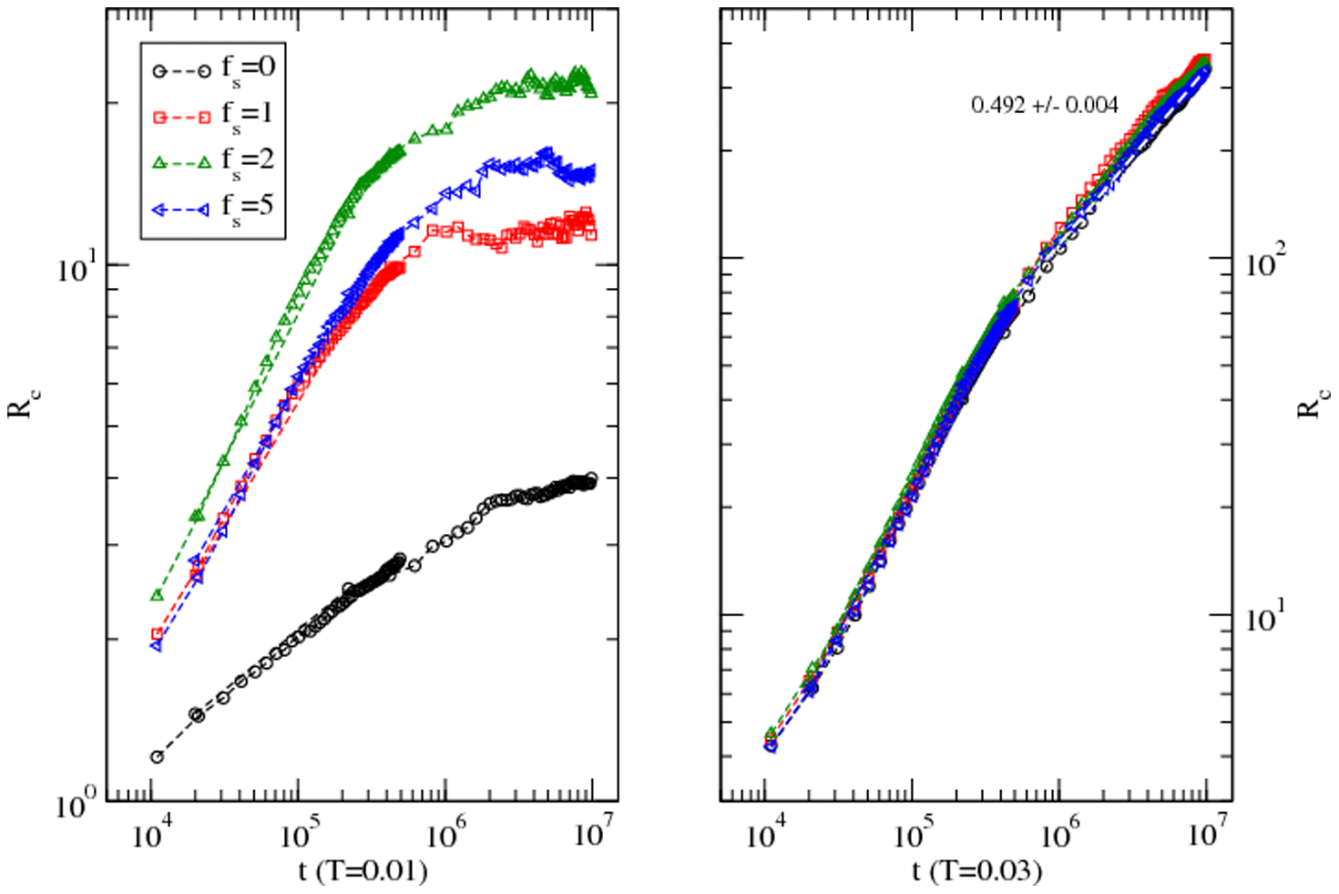
Variation of the root mean square displacement of the center of mass of the protein with the time step at a low (*T = 0.010*) and a high (*T = 0.030*) temperature in solvent with different interaction strengths (*f_s_ = 0–5*). Simulations are performed on a 64^3^ lattice with 100 independent samples at each temperature and solvent strength.

The global dynamics of protein and its form result from the cooperative and competing response of its residues as they perform their stochastic motion, settle, agitate, or simply trapped in its surrounding. We have analyzed the root mean square (RMS) displacement (*R_c_*) of the center of mass of the protein and its radius of gyration (*R_g_*) in detail in a range of solvent conditions and temperatures. Figure 4 shows the variation of the RMS displacements of the protein with the time step (*t*) at a low (*T = 0.010*) and a high (*T = 0.030*) temperature in different solvent conditions.

At the low temperature (T = 0.010), the protein moves rather fast initially (t≈10^4^–10^6^) before slowing down to a standstill in solvent with *f_s_ = 1–5* while it continues to move in the absence of solvent. Both residue-residue and residue-solvent interactions are affecting the dynamics of the protein. In the absence of solvent, the approach to the asymptotic slower dynamics of the protein is slower (with lower slope of the RMS displacement with the time step) than in the presence of the solvent. The interaction between the solvent and residues drives the protein to a faster equilibration to a quasi-static configuration. At the high temperature (T = 0.030), the RMS displacements approach diffusive dynamics (i.e. *R_c_∼t^1/2^*) when the solvent interaction becomes irrelevant. Temperature and interaction compete in orchestrating the dynamics; at such a high temperature thermal energy becomes dominant over the interaction leading to temperature-driven diffusion as expected. Although it is not fair to compare the RMS displacements of different proteins as a function of different variables such as time steps here and temperature in references 1(figure 1) and 10 (figure 1), its response to solvent and temperature seem consistent.

**Figure 5:**
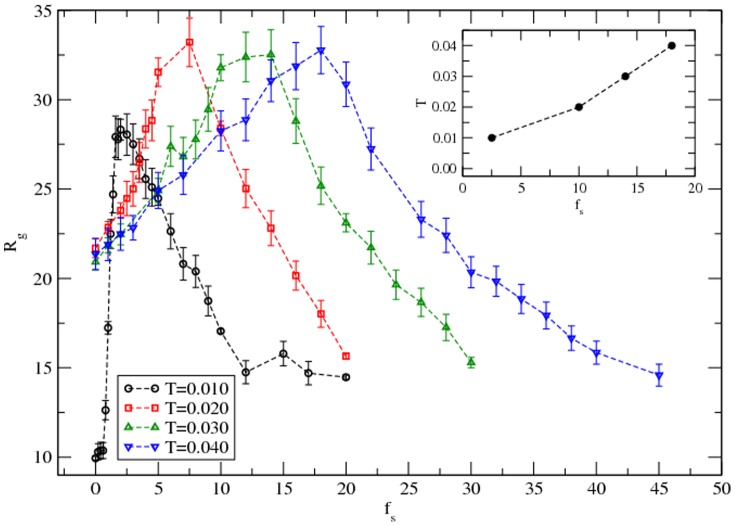
Variation of the radius of gyration of the protein with the magnitude of the solvent interaction at temperatures *T = 0.010–0.040*. Simulations are performed on a 64^3^ lattice with 100 independent samples at each temperature and solvent strength.

The radius of gyration (*R_g_*) is a measure of the size (resulting from the distribution of residues) of the protein. We have evaluated the radius of gyration in equilibrium in a range of solvent interactions at different temperatures. Variation of the radius of gyration with the solvent interaction is presented in figure 5.

At each temperature, the increase in the radius of gyration is followed by decay on increasing the magnitude of the solvent interaction. The radius of gyration responds non-monotonically to solvent interaction with a maximum at a characteristic value (*f_sc_*) at each temperature. The peak of the *R_g_* shifts towards higher solvent interactions on raising the temperature with broader distribution. Residue-residue and residue-solvent interactions are unique to each residue, which is distributed in a unique sequence in the protein (H3.1). The interplay between temperature and the push and pull due to interactions is rather complex. Nevertheless, the non-monotonic response of the radius of gyration with the solvent strength provides a general characteristic with a well-defined phase diagram (see inset in figure 5). The sensitivity of the dynamics and structural response of a different protein to solvent interactions has been recently studied by Miao et al. In addition to the unique specificity of the local properties, a protein (H3.1) may exhibit a general characteristic, e.g., non-monotonic response to solvent within the limitations of our model in this study. Unfortunately, we are not aware of any experiment (small or large scale) on histone H3.1 that can be explicitly compared to our data.

### Structure factor

As the name suggests, the structure function is a measure of structure, i.e., a way to quantify the distribution of residues of the protein. It is a Fourier transformation of the residue-residue correlation function and a useful quantity to identify structures as multiple length scales and is directly related to scattering experiments (e.g. neutron scattering) in probing the structures. The structure factor is defined as,
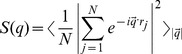
where *r_j_* is the position of each residue and *|q| = 2π/λ* is the wave vector of wavelength, *λ*, the length scale of the structure, i.e., spread of residues in the protein. If the structure factor exhibits a power-law scaling with the wave vector, i.e., *S(q) ∝ q^−1/ν^*, one can measure the spatial distribution of residues. For example, the radius of gyration (*R_g_*) of the protein is a measure of the linear spread of its residues, which shows a scaling with the number (*N*) of residues, *R_g_∝N^ν^*. Since the mass (of protein) is proportional to the number of its individual components (i.e., residues), *N ∝ R_g_^1/ν^*, which implies that the effective dimension of the protein *D_e_≈1/ν*.

**Figure 6:**
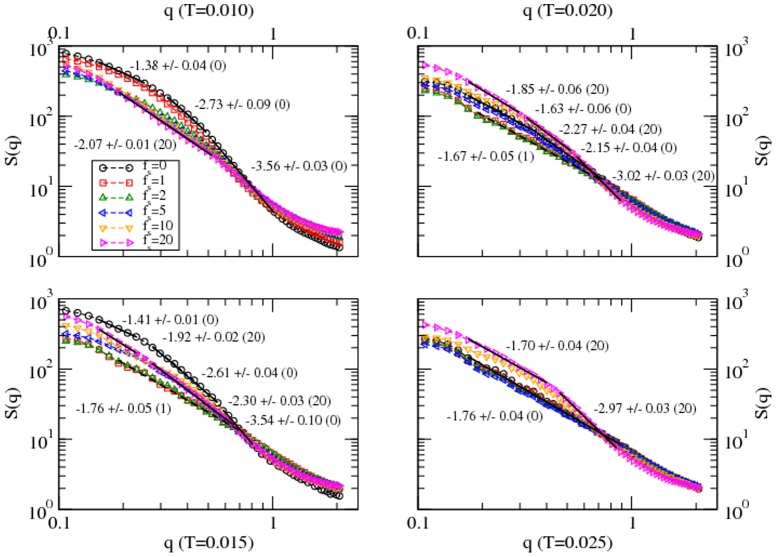
Variation of the structure factor *S(q)* of the protein H3.1 with the wave vector *q* in solvent with interaction strength *0–20* at temperatures *T = 0.010–0.025*. Slopes of the fitted data (covering the spread of the radius of gyration, see figure 5) are included with appropriate solvent interaction strength in parenthesis; the wave vector *q = 1* corresponds to a linear distance of 6.28 in units of lattice constant and *q = 0.1* to *62.8* (almost the entire lattice). Simulations are performed on a *64^3^* lattice with *100* independent samples at each temperature and solvent strength.

We have evaluated the structure factor *S(q)* in detail for a wide range of solvent strengths (*f_s_ = 0–20*) and temperatures (0.010–0.040). The variation of the structure factor for temperatures *T = 0.010–0.025* is presented in figure 6 to identify the trend. The magnitude of the slopes represents the effective dimension of the protein. The unsolvated protein coagulates to a globular structure at low temperature T = 0.010 with effective dimension representative of a solid, *D_e_∼3.00*. Increasing the temperature, the structure of the protein opens up and becomes a random coil (with exponent *D_e_∼1.76* of a self-avoiding walk (SAW)) at the high temperature *T = 0.025* (figure 6). In solvent with strong interaction (*f_s_ = 20*), the protein remains linear at low temperature, i.e., random walk *D_e_∼2.07* at *T = 0.010* and SAW *D_e_∼1.76* at *T = 0.015*. Raising the temperature to *T = 0.020*, the protein exhibits a local globular structure (*D_e_∼3.02* at high *q*) while retaining a chain morphology (*D_e_∼1.85* at low *q*), i.e., a linear chain of solid blobs. On further increasing the temperature to *T = 0.025*, we see that the structure of the protein, a chain of blobs (local assembly of residues) opens up by systematic decrease in its effective dimension. The quality of solvent orchestrates the multi-scale structure of the protein particularly at low temperatures. The specificity of the structural response is reduced dramatically at high temperatures. The response of the structure factor to solvent and temperature seem consistent with that of Sakai et al. (figure 3) in general; quantitative comparison is not feasible due to differences in proteins and scales including units.

### Fine-grained structure

**Figure 7:**
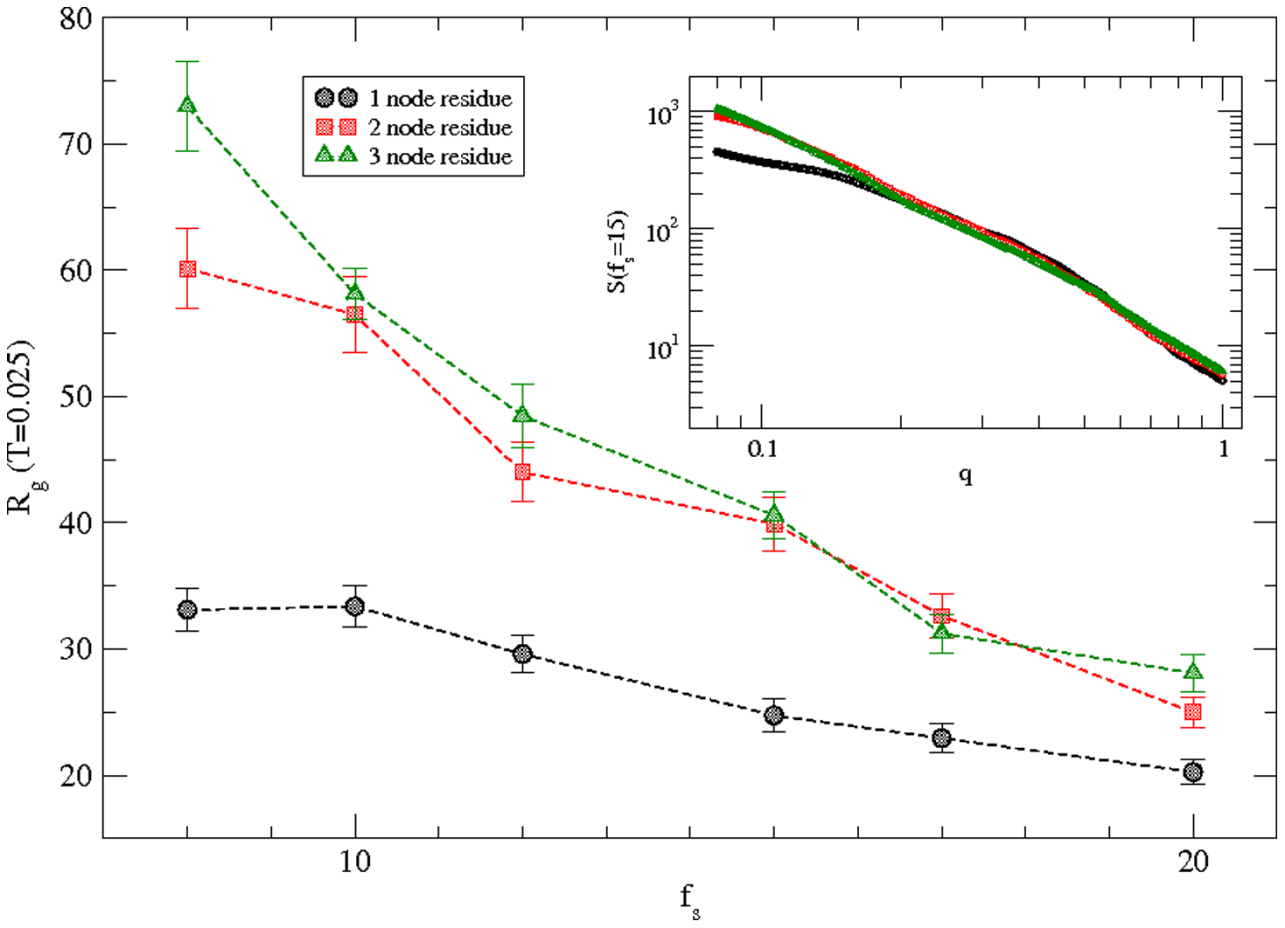
Variation of the radius of gyration of the protein with the magnitude of the solvent interaction at a temperature *T = 0.025* for protein h3.1 with multi-grain representations, i.e., one node, two nodes, and three nodes to represent each residue. Simulations are performed on a *64^3^* lattice with one node residue and on *100^3^* and *210^3^* lattices with two-node and three-node residues representations respectively. *100* independent samples with one-node, *50* with two-node, and *25* with three-node representations are used at each solvent strength. The inset is the structure factor *S(q)* versus wave vector *q* on a log-log scale at a representative solvent interaction strength *f_s_ = 15*.

Simulations are performed with fine-grain representation of the protein chain where a residue is represented by two and three consecutive nodes respectively. Variation of the radius of gyration of the protein with the solvent interaction is presented in figure 7 for the fine-grain protein along with original coarse-grain representation with one node per residue at a temperature *T = 0.025*. The radius of gyration of the protein in fine-grain representation is obviously larger due to higher number of nodes (*272, 408*) and chain lengths in comparison to original coarse-grained chain with only *136* nodes. However the monotonic decay of the radius of gyration with the solvent interaction strength remains qualitatively the same. Variation of the structure factor S(q) with the wave vector (*q*) is presented in the inset for a typical interaction strength (*f_s_ = 15*). Scaling of the structure factor with wave vector (*q*) remains the same over the length scale of the order of the radius of gyration of the protein. The large differences at large-scale (*q∼0.1*) between the *S(q)* of original coarse-grained chain and fine-grained chains are clearly due to differences in radius of gyration. The scaling extends for chains with larger lengths in fine-grained representation while it saturates for the original-coarse-grained chain at scales beyond its radius of gyration. Fine-grain representation provides better resolution in analyzing the structure of the protein. We hope to improve fine-graining by including the side chain of each residue [20] as the appropriate residue-residue interactions [21] become available.

## Conclusions

The effect of solvent on the structure and dynamics of a protein (H3.1) is studied at different temperatures from low to high values. The protein is modeled as a chain of residues tethered together via fluctuating peptide bonds with ample degrees of freedom on a cubic lattice where empty lattice sites act as solvent. A knowledge-based contact matrix (derived from an ensemble of protein structures in the PDB) is used as input to phenomenological residue-residue interactions. Each residue interacts with the solvent sites based on its hydropathy index; the quality of solvent is controlled by its interaction strength with the solvent via a parameter *f_s_*. Monte Carlo simulations are performed to study a range of local (e.g., energy and mobility profiles of residues) and global (e.g., RMS displacement of the center of mass of the protein, its radius of gyration, and structure factor) physical quantities. We find that the interaction between residue and solvent strongly affects both structure and dynamics of the protein particularly at low temperatures, which seem consistent with recent studies involving neutron scattering experiments and Molecular Dynamics simulations on different proteins. How does the protein structure evolution in a solvent depend on the temperature? For example, the radius of gyration (*R_g_*) of the protein H3.1 increases on increasing the solvent interaction strength until its characteristic value *f_sc_*, beyond which it decays, i.e., the response of the radius of gyration exhibits a maximum at *f_sc_*. The non-monotonic response of the radius of gyration to solvent interaction strength persists at each temperature, however, the characteristic solvent strength increases and the peak in variation of *R_g_* with the solvent interaction strength (*f_s_*) broadens with the temperature. Unlike a continuous mobile unsolvated protein, solvent immobilizes it at low temperatures. At high temperatures, the effect of the solvent becomes irrelevant as the protein dynamics become diffusive - a universal characteristic.

The analysis of the structure factor provides valuable insight into the multi-scale structures of the histone H3.1. Unsolvated histone H3.1 exhibits a continuous transition between a globular structure (at low temperature) to a random-coil configuration (ideal chain at high temperature) without segmental (i.e., local) self-assembly. Self-assembly of the residues (intra-protein) seem to persist in solvated histone; the size of globularity decreases on increasing the temperature. At each temperature (low to moderate), the multi-scale structural response of the protein in a solvent is unique and depends on the solvent interaction strength. The diversity in structure of the protein due to self-assembly of its residue in solvent preserves the specificity (interaction dependent structure) of the protein while it provides versatility in its functions. Unfortunately, we are not aware of experimental data on histone H3.1 at this time that can verify the consequences of such structural response, but we hope that this study may help with understanding future experiments.

Although, we cannot probe structure and dynamics of residues and protein at atomic scales due to limitations of our coarse-graining, we can analyze the segmental dynamics by examining the local quantities. Fine-grain representations of the protein are useful in gaining a better resolved structural detail while confirming the results of the original coarse-grained simulation qualitatively; we hope to improve fine-grain description in future. Interaction energy and mobility profiles of residues are useful in examining the segmental dynamics. The minimum energy of a residue does not necessarily mean lowest mobility as one may generally expect in purely interacting constituents. Because of the steric constraints of the peptide bonds and competing interactions and temperature, local frustrations cannot be ruled out in such a complex system. Our study may complement other investigations including those that are based on all-atom details in the ever growing interest in the field of protein modeling.
